# Supplementary figures and images for: Surveillance of Acute SARS-CoV-2 Infections in Elementary Schools and Daycare Facilities in Bavaria, Germany (09/2020–03/2021)
Source: Front Pediatr. 2022 Jul 6;10:888498. doi: 10.3389/fped.2022.888498 (PMC9298551; doi:10.3389/fped.2022.888498)

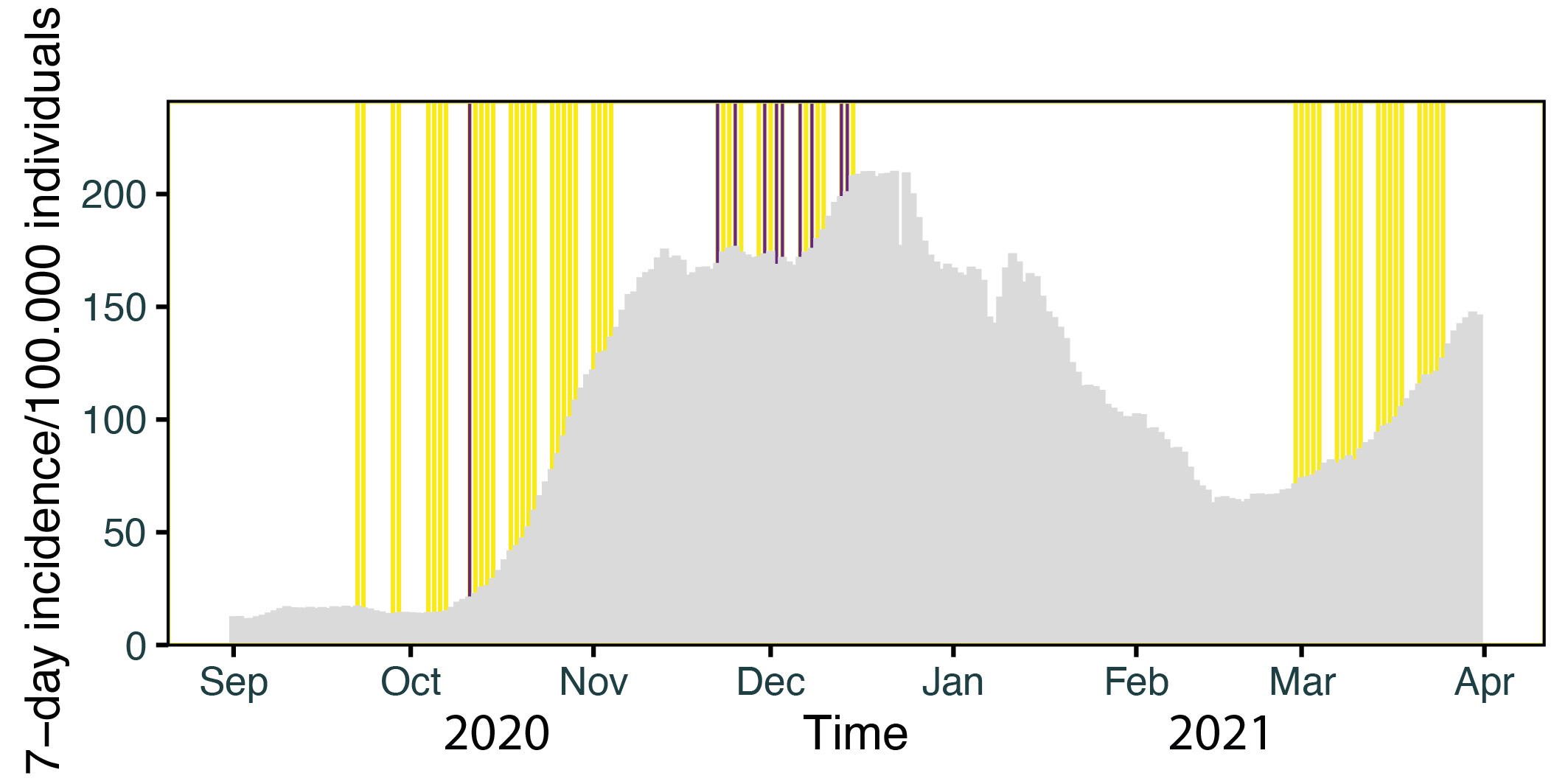

Supplement: Supplementary file 2 [file Data_Sheet_1.ZIP › supplementary material presentation/figure 4.jpg]

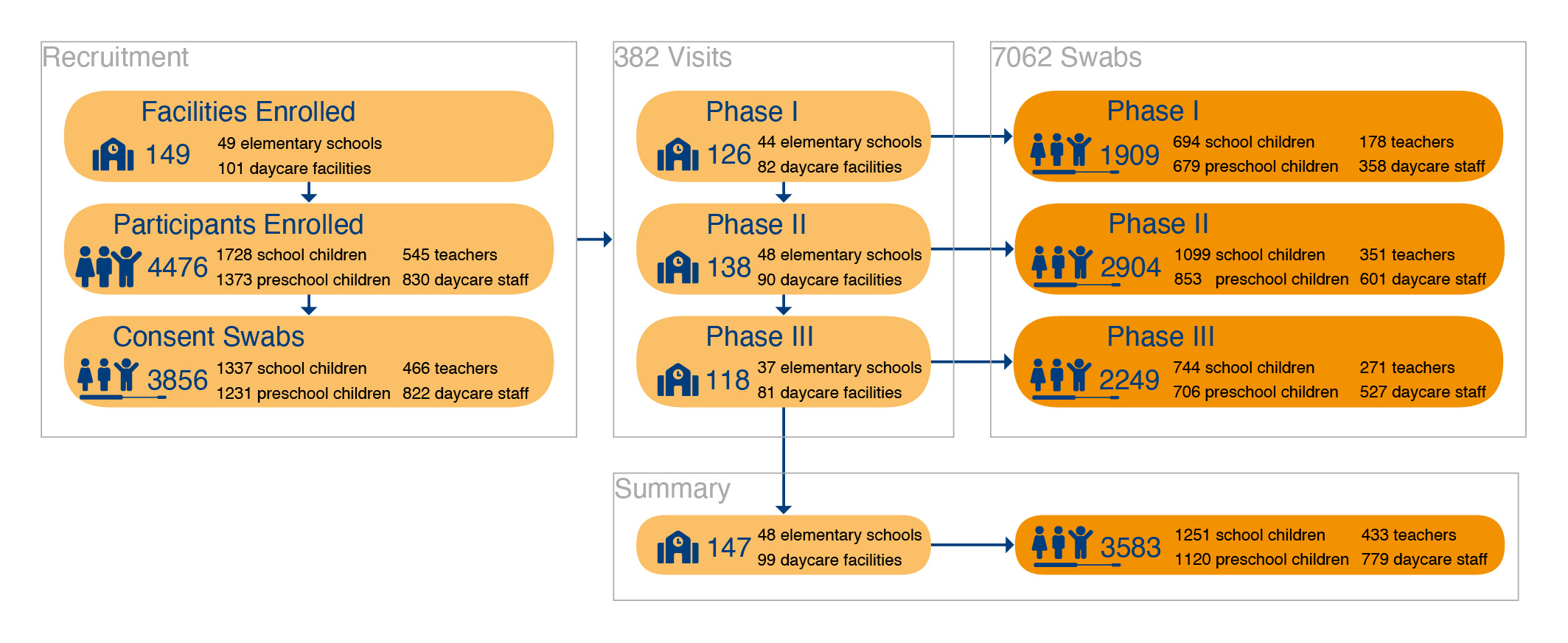

Supplement: Supplementary file 2 [file Data_Sheet_1.ZIP › supplementary material presentation/Figure2.jpg]
